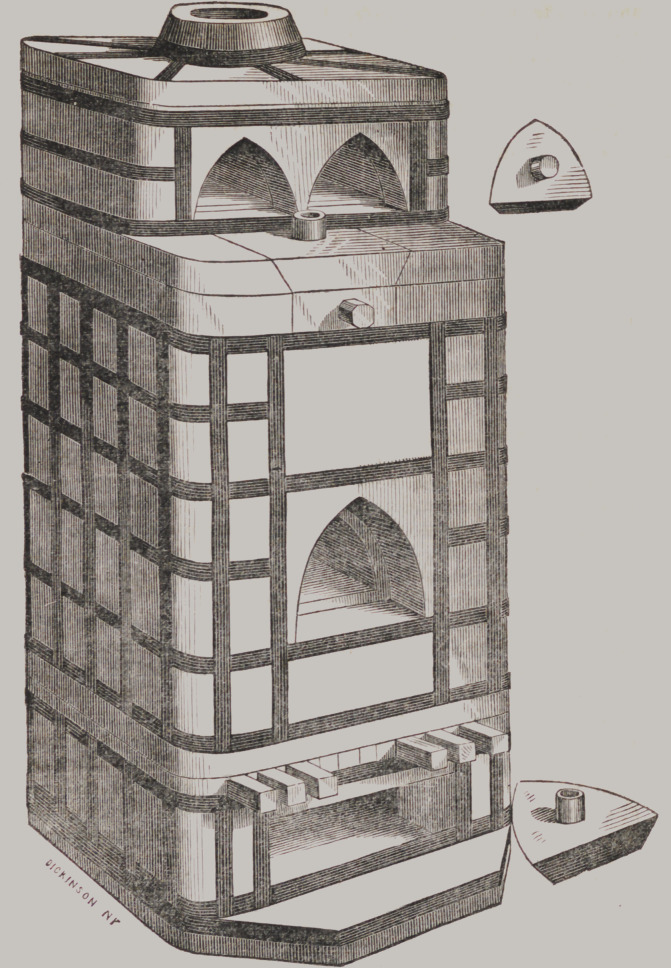# Introductory to Catalogue

**Published:** 1857

**Authors:** 


### INTRODUCTORY TO CATALOGUE.

In presenting the following Catalogue, the most complete of any before offered, I beg leave to say that I will make every effort to keep a constant supply of all the articles herein named, and add thereto all new and useful inventions, enabling Dentists to find at my establishment every article used by the profession.

*Manufacturing* my INSTRUMENTS and other Goods in large quantities, I am enabled to sell them at the lowest prices. Each article will be marked at a *fixed price*, from which I make no abatement, therefore, Dentists *can do as well* to ORDER by mail or express as by purchasing personally. In case I send anything not in accordance with the order, it may be returned and exchanged, or the money refunded, as may be requested. Every article sold by me is WARRANTED as represented.

Dentists about commencing business, as well as those replenishing, are solicited to call and examine my stock before purchasing elsewhere, as I am confident there can nowhere be found a better assortment, or as great inducements offered.

I have taken much pains in the arrangement of this Catalogue, giving illustrations of such things as would be difficult to understand. By preserving this Catalogue, and carefully referring to it, I trust it is so plain, that *definite* orders may be made out, and much labor of written description saved, and mistakes and delays avoided.

Very respectfully yours,
JNO. T. TOLAND.

### Gold Foil.

| | | |
|---|---|---|
| Jones, White & McCurdy's, - - | per oz. | $ 28 00 |
| J. B. Dunlevy's - - - | " | 28 00 |
| James Leslie's, - - - | " | 28 00 |
| Ashmead & Hurlburt's, - - - | " | 28 00 |
| David Morgan's, - - - - | " | 28 00 |
| Chas. Abbey & Son's, - - - | " | 32 00 |
| A. J. Watts & Co's Crystal Gold Foil, - | " | 32 00 |
| " " " Patent prepared Gold | " | 32 00 |

### Tin Foil.

| | |
|---|---|
| Jones, White & McCurdy's Chemically pure - | 75 |
| Griswold's, - - - - - | 75 |
| Kearsing's, - - - - - | 50 |

### Gold Plate.

| | |
|---|---|
| Gold Plate, 18 carats fine, (made from pure Gold,) per dwt. | 90 |
| Gold Plate, 18 carats fine, alloyed with Platina, suitable for clasps and backs, per dwt. - - - | 90 |
| Gold Wire, round and half-round, per dwt. - - | 90 |
| do. Spiral Springs, 18 carats, per pair, $2 50 to | 3 00 |
| do. do. 10 do. do. - - | 1 25 |
| Gold Solder, 18 carats, per dwt. - - - | 90 |
| do. 16 do. do. - - - | 80 |
| do. 14 do. do. - - - | 70 |

### Silver Plate.

| | | |
|---|---|---|
| Silver Plate, made from Coin, | - - per dwt. | 8 |
| do. pure, | - - - - - | 10 |
| Silver Wire, round and half-round, | - - - | 8 |
| Silver Precipitated, pure, per oz. | - - - | 2 00 |
| Silver Granulated, pure, do. | - - - | 2 00 |
| Silver Springs, per pair, | - - - - | 50 |
| Silver Solder, per dwt. | - - - - | 8 |

---

### Porcelain Teeth.

I have on hand and am receiving a large stock and splendid assortment of teeth, from the following celebrated manufacturers :

Jones, White & McCurdy,
N. Y. Teeth Manufacturing Co.,
Orum & Armstrong,
J. Klein.

My stock comprises the *finest selections* of all the best manufacturers, which I am prepared to sell at the following *very low prices :*

| | |
|---|---|
| Gum Front Teeth, each, - - - | 20 cents. |
| Gum Mo. and Bi. Teeth, each, - - | 20 " |
| Plain Front do. do. - - | 10 " |
| do. M. and B. do. do. - - | 10 " |
| Continuous Gum Teeth, do. - - | 10 " |
| Teeth for Gutta Percha work, each, - | 10 " |
| Pivot Teeth, - - - - | 8 " |

I give a discount of 3 per cent. on bills of $30 worth of teeth, 5 per cent on bills of $50, and 10 per cent. on $100.

☞ *Dealers* will be supplied on liberal terms.

☞ Teeth carefully selected to casts, or according to samples and directions, without extra charge.

---

### Block Work.

Executed in the most beautiful and artistic style *exclusively* for the Profession.

| | | | |
|---|---|---|---|
| Block Work carved and fitted at | 50 | cents per Tooth. | |
| " " and mounted | $1 25 | " | " |
| Continuous Gum Work . . | 1 25 | " | " |
| Single Gum Teeth mounted for | 1 25 | " | " |
| " Plain . . . . | 1 00 | " | " |
| Gutta Percha Work . . . | 75 | " | " |

Every dentist must furnish his own materials, or if desired, I will furnish at the regular prices. The above are the prices for full arches, or nearly so. Cases of less than six teeth will be charged 25 cents per tooth, extra.

---

### Platina Imported.

| | |
|---|---|
| French Plate at $7 50 per oz. | French Wire at $7 50 per oz. |
| English Plate. | Re-melted Plate, 7 50 " |

Platina Plate *cut to pattern* at $8 75 per oz.

Thin plate, in strips, for batteries.

The English Platina costs from twenty-five to fifty cents less than the French and re-melted, but is not reliable; therefore I do not keep it on hand, but will furnish to order.

Platina rolled to any desired thickness, without extra charge.

☞ Pure Gold, for soldering Platina, at $1 20 the dwt.

☞ Gold, Silver, and Platina *Scraps* taken in exchange for goods.

---

### Sherwood's Forceps.

These are manufactured by Mr. H. R. SHERWOOD *exclusively for my sales* according to the most approved patterns, *perfectly* adapted to the *Hand* and *tooth*, enabling the Dentist to operate with *ease* and *certainty*.

☞ N. B. We regret to state in this connexion that there are Dealers who deem it to *their interests* to endeavor to bring our instruments into disrepute, by having the same shaped instruments made by *Inferior workmen*, and then advertising "Sherwood's Patterns" at prices far below our prices, and as much below what they sold them at whilst *they had them* for sale.

We will however trust to the *honor* and GOOD SENSE of the Profession (which thus far has not failed) to discriminate between, the GENUINE article which has received the sanction of their universal approval—and an IMITATION of (to say the least) *Doubtful* quality.

☞ Whenever a Dutchman ("over the Rhine") can be found to make Forceps EQUAL in any respect to those made by Mr. Sherwood, we will then think of entering into competition, but until then, those willing to be deceived, are welcome to their BARGAINS.

The following is a list of our regular Shapes of which we endeavor to keep a constant supply on hand, although at present we have *Orders* for more than we can make in the next two months, but we are endeavoring to get additional hands and extend our facilities for manufacturing.

The prices are the same throughout, regardless of shape. Any other patterns will be made at the same prices, as follows:—

| | | |
|---|---|---|
| Broad Fancy Handles, octagon joints and jeweled, | each, | $3 50 |
| “ “ “ “ “ without “ | “ | 3 00 |
| Broad plain “ “ “ Jeweled, | “ | 3 00 |
| “ “ “ “ “ without “ | “ | 2 25 |
| Narrow “ “ “ “ like Eastern | “ | 2 00 |
| Broad Oval jointed Forceps, first quality, | “ | 2 00 |
| Narrow “ “ “ like Eastern | “ | 1 75 |
| “ “ “ “ 2d quality, | “ | 1 50 |

Forceps for the Superior Dentes Sapientia.
“ “ “ “ “ “ Roots.
“ “ “ Inferior “ “
“ “ “ “ “ “ Roots.
“ “ Elevating the roots of “
“ “ the Superior Molars, Right Side.
“ “ “ “ “ Left “
“ “ “ “ “ “ “ Roots.
“ “ “ “ “ Right “ “
“ “ “ Inferior “ “ “
“ “ “ “ “ Left “
“ “ “ “ “ Either Side.
“ “ “ “ “ Roots.
“ “ “ Superior Bicuspids, Either Side.
“ “ “ Inferior “ Right “
“ “ “ “ “ Left “
“ “ “ “ “ Either Side.
“ “ “ Superior Incisors, “ “
“ “ “ Inferior “ “ “
“ “ “ Superior Roots.
“ “ “ Inferior “
“ Narrow Beak Hawk's Bill for crowded Teeth.
“ for Separating Roots of Teeth.
“ “ Excising Teeth Straight.
“ “ “ “ Curved.

Light Forceps for Children's Teeth at corresponding prices.
Prof. Harris' patterns of Forceps.
“ Taylor's “ “ “
Dr. W. M. Hunter's patterns of Forceps.

| | |
|---|---|
| Dr. Elliot's Pivot Extractor, . . . . . | $ 3 00 |
| “ Hullihen's Screw Forceps, . . . . . | 2 50 |
| “ “ “ “ with Chevalier's Improvement, | 3 00 |
| “ Baxter's Plugging Forceps, plain, . . . | 5 00 |
| “ “ “ “ Pearl sides, . . | 8 00 |

### Forceps for Physicians.

Shaped to the Tooth and hand, made of good metal and neatly finished.

Upper Molar, with curve or hook for little finger, Either Side.
" Bicuspid, " " " "
" Incisor, Straight.
Lower Molar and Dentes Sapientia, Either Side.
Lower Fang, curved.
Upper Fang, straight.

Price of each, $ 1 25.

*Also,*

Common Forceps, consisting of straight and curved.

| | | | |
|---|---|---|---|
| Single jointed, | | . . . . . | 60 cents each. |
| Double " | small | . . . . | 75 " " |
| " " | large | . . . . | $1 00 each. |

A Liberal Discount to Dealers who purchase by the dozen to sell again.

---

### Chevalier's Forceps and Instruments.

I am prepared to supply the profession with every kind and description of Instruments, manufactured by Mr. J. D. Chevalier, for the same prices at which they are sold at his establishment in New York.

*Also,*

H. G. Kern's instruments, at his prices.
J. H. Gemrig's instruments, "
And all others at the respective manufacturer's prices.

---

### Lancets.

| | |
|---|---|
| Ivory handles, flat, . . . . . . . | $ 50 |
| " " oct. silver ferrule, . . . . . | 75 |
| Pearl " . . . . . . . . from $2 to | 3 50 |
| Additional for a revolving blade, . . . . . | 50 |
| Pocket lancets, one blade, . . . . . . | 50 |
| " " " spring, . . . . . | 1 50 |
| " " two " . . . . . . . | 1 75 |
| " " three " . . . . . . . | 2 00 |

### Plugging Instruments.

| | | | |
|---|---|---|---|
| Bone twist handles, albata ferrules, . | | per doz., | $6 00 |
| With ¼ inch steel handles, file cut octagon | | „ | 4 00 |
| " ⅜ " " " " " | | " . | 6 00 |
| " ½ " " " " " | | " . | 7 50 |
| " ½ " ivory octagon handles, sterling silver ferrule, | | | 9 00 |
| " ⅝ " " " " " " | | " | 12 00 |
| " ¾ " " " " " " | | " | 16 50 |
| " ⅝ " ebony " " " | | " | 9 00 |
| " ¾ " " " " " " | | " | 12 00 |

The above have broad and heavy *GENUINE SILVER FERRULES*; those desiring albata (German silver,) ferrules, can have them at a corresponding reduction in price.

| | |
|---|---|
| With octagon pearl handles, gold ferrules, | $24 00 to $36 00 |
| " flat pearl handles carved and jewelled, | $40 00 to $50 00 |
| " Cameo handles, gold ferrules, | $24 00 to $45 00 |

---

### Scaling and Cutting Instruments.

With steel, ebony, ivory, pearl or cameo, of various styles and prices, same as pluggers.

---

### Excavators, Burs and Drills.

| | |
|---|---|
| With round wire handles, per dozen, . . . . | $1 00 |
| " smooth octagon wire handles, . . . . | 1 50 |
| " octagon file cut " . . . | $2 00 to $4 50 |
| " ⅜ inch ivory, file cut, silver ferrules, . . | 6 00 |
| " pearl handles from . . . | $12 00 to $24 00 |

---

### Turnkeys.

| | | | |
|---|---|---|---|
| Straight Shaft, | $1 00 | Clark's Turnkey, | $1 75 |
| Spring Bolt Turnkey | 2 00 | English " | 1 50 |

---

### Elevators.

| | |
|---|---|
| Hook shaped, | Spoon shaped, |
| Punch shaped, | Push and Pull shaped, |
| Right and Left shaped, | Kentucky Hook shaped, |

Screws and all other shapes,

| | |
|---|---|
| With Ebony Handles . . . . . | 50 cts to 75 cts. |
| " Ivory " . | $1 00, $1 25, $1 50 and $1 75 |
| " Pearl " . . . . . . | $2 00 to $4 00 |

### Mouth Glasses.

| | |
|---|---|
| Pearl Gold Mounted with joint and sets, . . . | $5 00 |
| " " " Dolphin pattern, . . . | 2 25 |
| " " " " star " . . . . . | 2 50 |
| " Silver " carved and joint, . . . | 2 00 |
| " " " plain " . . . | 1 75 |
| " " " Dolphin . . . . | 1 50 |
| " " " plain for pocket, . | $1 00 to 1 25 |
| Ivory, plain, for pocket, . . . . . . | 60 cts. |
| Ebony, " " " . . . . . . . . | 40 " |
| Rosewood and Mahogany, . . . . . . | 25 " |

### Hand Mirrors.

| | |
|---|---|
| Pearl, Silver Mounted, . . . | from $5 00 to $10 00 |
| " Gold " . . . . . . | " 7 00 to 15 00 |
| " " " with settings . | " 10 00 to 50 00 |
| Rosewood, . . . . . . | 37½ cts. to $1 00 |

### Syringes.

| | |
|---|---|
| Gold, a very handsome article, . . . . . | $15 00 |
| Silver, . . . . . . . . . | 5 00 |
| Syringe metal, with Silver point, . . . . . | 75 |
| Glass, plain, . . . . . . . . | 25 |
| Gutta Percha, . . . . . . . . . | 1 00 |

### Files.

EARNEST'S, MURPHY'S, AND OTHERS.

| | | |
|---|---|---|
| Separating, . . . | per dozen, | $1 00 or 10 cts. each. |
| " Bevel Edge, . | " | 1 75 & $2 or 15 and 18 |
| " Flat, oval, blunt and pointed, | | 1 75 & 2 25 or 15 & 20 |
| Molar, single curve, . | per dozen, | 2 25 or 20 cts. each. |
| " double " . . | " | 2 50 or 25 " |
| " " " Feather edge, | " | 3 00 or 30 " |
| Oval Stump, . . . . . | " | 2 50 or 20 and 25 |
| ½ Round Stump, . . . . . . | | 2 50 or 20 cts. each. |
| Plug for lateral cavities, of various patterns, . . . . . | " | 2 25 to 2 75 or 20 to 25 |
| Plug for crown cavities, of various patterns, . . . . | " | 2 25 to 2 75 or 20 to 25 |
| Bicuspid, thin and thick, . | " | 2 25 or 20 cts. each. |
| Plate, of various patterns, . | " | 2 50 or 25 " |
| " for filing solder, . . | " | 2 50 or 25 " |

STUBS'.

| | | | |
|---|---|---|---|
| Separating, | | per dozen, | $1 50 |
| Bastard, smooth and rough, | 6 in., | each | 30 cts. |
| " " " | 5 in., | " | 25 cts. |
| " " " | 4 in., | " | 22 cts. |
| " " | 3 in., | " | 18 cts. |
| " " assorted. | | | |

---

### Dental Cases.

12 pair Extracting Forceps, Sherwood's octagon.
2 large Ebony Screws.
1 " Ivory Elevator.
1 dozen Ivory Handled Pluggers, Silver Ferrules.
1 " " " Cutting and scaling Instruments, with Silver Ferrules.
1 dozen octagon Steel Handled Pluggers.
2 " Wire " Excavators.
2 " " Drills.
1 Ivory Socket with revolving head, and one dozen Drills.
1 pair Steel Foil Scissors.
1 Pearl Hand Mirror.
1 " Mouth "
1 " Cheek Holder.

Rosewood or Mahogany Case, Brass bound, with drawers for Forceps, and trays for the instruments, and space beneath the trays divided into apartments for Teeth, foil, &c., handsomely lined with silk velvet.

The above is what is recommended by the FACULTY of the Ohio College of Dental Surgery to the *Graduates*, as an outfit containing what is necessary, and nothing superfluous, filled with Sherwood's instruments got up in good style. The cost is $100.

The same instruments and case may be furnished from $75 to $150, by varying the style and quality. Of these we make three sizes, viz: 16 inches, 18 inches and 20 inches long. Prices, $12, $14 and $16.

Fitting and trimming with fine silk velvet, $15, $20 and $25.

Fitting and trimming with cotton velvet, $12, $14 and $16.

This illustrates a very handsome style of case, termed a "Five Drawer Case." Price according to quality and number of instruments, from $125 to $500.

Of these we make five sizes, 16 inches, 18 inches, 20 inches, 22 inches, and 24 inches LONG, width and height in proportion. Prices of empty cases of this kind from $15, $18, $20, $23, $25. Fitting & Trimming with fine silk velvet, $15, $20, $25, $30, $35.

A gentleman wishing to order a set of Instruments, should begin, say with the kind and size of case, and how to be trimmed, whether with *silk* or *cotton velvet.* Next, Forceps, specifying the kinds, quality and shapes. Next, pearl mirror, mouth mirror, foil scissors, scalers, plugers, syringe, lancets, etc,, etc., being specific as to kinds, styles and how many of each. List of prices will be found in another part of this work. Any style and price of case got up in the neatest manner, at corresponding low prices.

As may be supposed, in ordering a case, any alterations may be made in the arrangement of instruments. Some will want less Forceps, and more of other Instruments than others. In all cases, they will be put up according to instructions given, and at uniform rate of charges. In other words, I do not wish any one to adopt my plan of arrangement (good as I deem it, from much experience in this line), but hope each one will use his own judgment, and order accordingly, depending upon me to *carry out* their wishes *faithfully.*

The *demand* for *fine* Goods is rapidly increasing, so far beyond our anticipations, that we have been compelled to delay and disappoint many of our best friends. We may safely assert that at least *five* times as many "FIRST CLASS" Instruments have been sold in Cincinnati within the last year, than during any previous year.

I must ask a little *patience.* I am making arrangements to extend our manufacturing facilities, and hope soon to be able to procure competent hands to meet the increased demand.

By sending in your orders some time in advance, I hope to be able to supply all in good time. It is better to do this, and get just what you want of reliable instruments, than to pick up an outfit promiscuously of inferior Goods, and at greater expense.

☞ In a *very short* time, I will have arrangements completed to fill all orders with the least possible delay.

---

### Dental Chairs.

| | |
|---|---|
| Mahogany, Movable Seat, Back, and Head-piece, large Wheel and Screw to raise the Seat, covered with Plain or Figured Plush, . | 55 00 to 65 0[illegible] |
| Walnut, do. do. do. do. do. | 55 00 to 65 0[illegible] |
| Mahogany, Movable Seat, Back, and Head-piece, | 45 00 to 55 0[illegible] |
| Walnut, do. do. do. | 45 00 to 55 0[illegible] |
| Mahogany, Movable Seat and Head-rest, | 40 00 to 45 0[illegible] |
| Walnut, do. do. do. | 40 00 to 45 0[illegible] |
| Mahogany, Stationary Back and Seat, Movable Head . . . . . . . | 35 00 |
| Walnut Barbers' Chairs, . . . . | 20 00 & 25 00 |
| Chairs after Justus Ask's patent, from | 35 00 to 85 00 |
| Perkins' Patent Ball and Socket Chairs—Mahogany, . . | $100 |
| " " " " " " Rosewood, . . | 125 |

Having many inquiries in reference to dental chairs, we give the above cut, of the kind we get up, and have for sale. They are made of mahogany or black walnut, as may be desired, covered with scarlet figured plush. The seat being made to raise by a crank extending out from under the chair, as may be seen ; thus, any elevation of the seat may be obtained. The back falls by means of two quadrants, both being controlled by moving one ; thus obviating the necessity of going behind the chair to let the back down. The head-piece raises or falls by means of a quadrant, and a part of the headpiece slides upwards and downwards, and *horizontally ;* the latter movement enabling the operator to bring the head of the patient close to his breast, by which he may operate without stooping. They are very strong and handsome, and combine all that is requisite in a dental chair.

We also make two others a little different. One with the back permanent, but all the other movements as above described; another and a cheaper kind, with back and seat permanent, but movable head-piece.

Almost any taste can be suited.

This chair differs from the preceding, only in the apparatus used for raising the seat, which is clearly shown in the cut. It is powerful, and every way efficient. The view shows the lateral movement of the head-piece, which has also an upward movement.

Price, $55 to $65. Boxing, $2.50 extra.

This newly invented and arranged operating chair, after Justus Ask's patent, combines all the best principles known, and more of them than any chair heretofore made, and at a much lower price.

The head-piece rises and falls perpendicularly, and moves backwards and forwards with a circular ratchet. The seat rises or lowers by a crank, placed either at the side or back (works with equal facility with the patient in or out of the chair) by means of a stationary double-threaded screw, remaining in the position it is turned to without any fastening.—The back and body of the chair can be pitched backwards or forwards, (carrying the seat with them), by a foot-lever, and stopped at any desired angle. The entire apparatus of this chair is simple, strong and effective. The seat moving with the back will be found of great advantage, preventing the annoyance of patients constantly sliding away from the operator. We have a good assortment of these chairs, from $40 to $86; boxing, packing and porterage, $2.50. The working principles and raising and lowering apparatus are the same in all the chairs.

### Tooth-Powder Boxes.

| | |
|---|---|
| Wooden, No. 1, plain tunred, | 20 to 30 |
| do. " 2, . labelled, | 50 |
| do. " 3, | 60 |
| do. " 4, | 70 |
| Glass, with Metallic tops, | 75 to 1.00 |

### Spittoons.

| | |
|---|---|
| Mahogany, with Octagon Marble Top, Octagon Base and Column, | $15 00 |
| Walnut do. do. do. do. do. | 15 00 |
| Mahogany, with Square Marble Top, Square Base and Column, | $10 00 to 12 00 |
| Walnut, do. do. do. do. | 10 00 to 12 00 |
| Boxing for shipment, extra. | |
| Brass Crane, with Bowl and Funnel to attach to Chair, | 8 50 |
| Brass Crane and Table to attach to Chair, to lay instruments upon whilst operating, | 8 50 |

### Rolling Mills.

| | |
|---|---|
| 3 Inch plain bolted on neat Iron Columns,. | 35 00 |
| 3 " Geared " " " | 40 00 |
| 3½ " Plain " " " | 40 00 |
| 3½ " Geared " " " | 45 00 |
| 4 " Plain " " " | 45 00 |
| 4 " Geared " " " | 50 00 |
| Extra (or Double) Geared, 3½ in. | 85 00 |
| Do. " " 4 " | 90 00 |
| Do. " " 4½ " | 100 00 |

Without the Iron Column, a deduction of $5 will be made from the above prices.

These Mills are made by Helm & Dorance, and are emphatically the best Roll in the market, and are warranted in every case.

### For Mechanical Dentistry.

| | |
|---|---|
| Acid Pans, Copper, for heating acids for pickling, | 75 |
| Anvils, | 75 to 1 00 |
| do. in lead bases, | 1 25 to 1 50 |

Arkansas stones for Lancets, Excavators, Gravers, etc., . . . . . . . from 75 to 4 00
Arkansas stones, for dressing fillings, . " 75 to 1 00
Articulators, . . . . . . . " 1 00 to 1 25
Blow-pipes, Brass, for Mouth, 9 inch, . 25
do. do. 10 do. . 30
do. do. 11 do. . 35
do. do. 12 do. . 40
do. do. 13 do. . 45
do. do. 14 do. . 50
do. Steam self-acting . . . . 3 50
do. " Copper Boiler, . 5 00
do. " Brass " . . 6 00
do. " do. (Macomber's patent improved), 10 00
do. " Steam Self-acting brass boiler, 10 00
do. " do. (O. L. Lawson's greatly improved, with gauge cock, to control the flame 12 00
Burnishers for plates, Steel, . . . . . . 40
do. do. Blood stone, . . . 1 00 & 1 25
Burrs for trimming off superfluous solder, . 50
Crocus, per box, . . . . . . . 12
Corundum Wheels, No. 00, each, . . . 10
do. " 0, " . . . 10
do. " 1, " . . . 12
do. " 2, " . . . 15
do. " 3, " . . . 18
do. " 4, " . . . 20
do. " 5, " . . . 25
do. " 6, " . . . 30
do. " 7, " . . . 40
do. " 8, " . . . 50
do. " 9, " . . . 75
do. " 10, " . . . 1 00
Corundum Cones,. . . . . . . . 15
do. Slabs, . . . . . . . . 40
do Files, round, taper and flat oval, . . 25
Draw plates, various sizes, . . from $1 12 to 4 50
Drill Stooks, Brass, small, . . . " 50 and 75
do. do. . . . . . 1 50
Grinding Apparatus, Iron Painted, accelerated motion to run 1 wheel, . . . . . 2 75
do. do. Iron, accelerated motion open, . 3 00
do. do. do. do. with band, . . . 6 50
Hammers for riveting, without handles, . . 35
do. do. do. Stubs' . . 40
do. do. with handles, . from 40 to 50

| | | |
|---|---|---|
| Hand Brushes, Jewellers', | | 25 |
| do. Plate, | | 35 |
| Handles of wood, for files, etc. | from 4 to | 10 |
| Impression Cups, Tin, | | 15 |
| do. Britannia metal, various patterns, for plaster or wax, | | 40 |
| do. Britannia, for taking impressions of lower jaw, in plaster or wax, | | 75 |
| Ingot moulds, Iron, plain, to slide, | | 1 25 |
| do. do. do. with clamp, | | 1 75 |
| do. Iron, with broad base, to slide | | 2 50 |
| do. Soapstone, | | 75 |
| Lamps for soldering, for oil, | from 50 to | 75 |
| do. do " alcohol, | " 50 to | 75 |
| do. Taft and Watt's. | | |
| Ladles of Cast Iron, with Wrought Iron handles, for melting Zinc and Lead, . No. 5, | | 50 |
| do. do. do. do. do. " 6, | | 60 |
| do. do. do. do. do. " 7, | | 70 |
| do. do. do. do. Shifting Handles, | | 75 |
| Lathes, to be driven by the foot, Chevalier's, | | 15 00 |
| do. do. do. Heavy Wheel, | | 16 00 |
| Moulding Flasks (Hawes') | | 2 00 |
| do. (Locke's), eight pieces, | | 2 00 |
| do. (Luther's) | | 1 00 |
| Magnets for removing Iron or Steel from fillings, | 50 to | 1 00 |
| Nippers, front, | 50 to | 75 |
| do. side, | 60 to | 75 |
| Forceps for Bending Plates, black, | | 1 50 |
| " " " " oval polished, | | 1 75 |
| " " " " oct. " | $2 00 and | 2 25 |
| " " " Clasps, black, | | 1 00 |
| " " " " oval polished, | | 1 25 |
| Plate Cutters for cutting out plate in fitting around the teeth, | | 1 50 |
| " Punch, plain black, | | 1 50 |
| " " " oval polished, | | 1 75 |
| " " octagon " | 2 00 and | 2 25 |
| " " Chevalier's improved, black | | 2 00 |
| " " " " polished, | | 2 50 |
| " " Mallett's adjustable to punch both holes at once, | | 5 00 |
| Plate Shears, for cutting plate, Stubs', straight, | from 60 to | 1 00 |
| do. do. do. curved, | " 75 to | 1 25 |
| Plate Gauges, round, American, | | 1 00 |
| do. do. Stubs', | | 2 50 |

| | |
|---|---|
| Rotten Stone, pulverized, for polishing Gold, per bot., | $ 50 |
| Rouge, for polishing Gold, . . . . per box, 25 and | 50 |
| Saw Frames, . . . . . . each, | 75 |
| Saws for Frames, . . . . . . per doz., | 25 |
| Saws, small circular, for cutting Teeth off plate, each, | 50 |
| Scales and Weights, . . . . . 75 to 1 00 to | 10 00 |
| Screw plates and taps, . . . . . 75 to | 2 00 |
| Scrapers for cleaning Plates, . . each, 25, 35 and | 40 |
| do. do. triangular, American, " | 50 |
| do. do. do. Stubs', " | 75 |
| Scotch stones, . . . . . . from 10 to | 25 |
| Soldering Pans, . . . . . " 75 to | 1 00 |
| Stakes, to set in Wood, or fasten in Vice, per ℔, | 50 |
| Slayton's Colored Gutta Percha Base, per ¼ ℔, | 5 00 |
| Spatula's, for working Slayton's Material, . . | 50 |
| Spring Dividers, . . . . . . 50, 62 and | 75 |
| Tongs, for Ingot moulds, . . . . . . | 50 |
| Teeth holders, for holding Teeth, while grinding, . | 31 |
| Tweezers or Spring Forceps, for picking up Solder, | 20 |
| do. do. do. do. from 6 to 12 inches long, | 50 |
| do. do. curved points, fine finish, | 50 |
| Plyers, round and flat-nosed, American, . . | 25 to 35 |
| do. do. Stubs', . . | 40 to 60 |
| Vices, for Bench, . . . . from $1 25 to | 10 00 |
| do. Pin, with Screw, . . . . . 75 to | 1 00 |
| do. do. Slides, . . . . . . 75 to | 1 00 |
| do. do. do. small, . . . . . | 50 |
| do. Hand, with Screw, . . . . . . 75 and | 1 00 |
| Wheel Brushes, hard and soft, 2 rows, each, . . | 35 |
| do. do. 3 " " . . | 40 |
| do. do. 4 " " . . | 50 |

---

### Saliva Pump.

This little article was designed by Professor Arthur, for removing the saliva from the mouth, so necessary while filling the lower molars. It is all of glass, excepting the large bulb, which is gum-elastic. It is applied by inserting the small point under the tongue, and by pressing upon the gum bulb the air is driven out, and on relieving the pressure, the vacuum thus created is filled by drawing the saliva into the centre bulb. Price $1.

### Macomber's Gas Blow-pipe.

This is an appliance designed to be attached to a gas pipe, for using gas instead of oil or alcohol. No. 1, is a double tube, or rather a tube enclosing a tube, the atmospheric air being driven through the centre tube, adding force and giving a cylindrical form to the flame. No. 2, is a stop-cock by which the size of the flame may be regulated. No. 3, is a movable joint, by which the flame can be directed upward or downward. They work admirably, and are much more economical than alcohol or oil lamps. Price $3 50.

We have, also, this gentleman's "Patent Improved Alcohol Blow-pipe," greatly modified and improved from the style in which it was originally brought out. Price $10.

---

### Self-Acting Blow-pipe.

Is manufactured wholly of brass, with the exception of the pedestal which is of iron. The ball is thick and strong, with safety valve on top, and every part constructed in the most substantial and workmanlike manner, being made by an excellent artizan of long experience in his business. To persons having feeble lungs, or otherwise disinclined to use the mouth blow-pipe, this apparatus is an invaluable aquisition. Price, $10 00; extra thickness, $10 50.

The Company has also the Bellows Blow-pipe, constructed by Dr. Loomis, of Saratoga, impelled by a lateral motion of the knee. Price $10 00.

### Lawson's Patent Alcoholic Self-acting Blow-pipe.

This Blow-pipe is entirely new, and the most complete yet brought before the Profession, a description of which must satisfy any one of its superiority.

A, A, are screws to control the safety valve B, by which more or less power is obtained.

C is the boiler.

D is a *gauge cock* which controls the discharge of the vapor from the pipes, and here is the great improvement. The objection heretofore against Self-acting Steam Blow-pipes, has been the want of control over the discharge of the vapor, but by this arrangement perfect management of the force or power is obtained, and not only this, but the jet may be changed at pleasure from one pipe to the other—from a large flame to a fine jet, and all by this gauge cock.

E, F, are where the pipes are screwed to the tubes in the boiler, and can be removed at pleasure, for the purpose of cleaning or the substitution of others.

G and H, the two points of the pipes—large and small.

K, a screw to tighten the upright in the base, which upright is movable in the socket.

L, another screw to tighten the arm on which the small lamp M, rests, and which slides upon the upright. This small lamp has a movable tube at N, by which the wick is raised or depressed. So also the large lamp at O. This arrangement is useful in affording facility for increasing or decreasing the flame at pleasure.

It will now be seen that this Blow-pipe is just as managable as the lungs, and far more powerful, therefore more efficient, which will be found to be true on trial.

Price to Dentists, $12 00.

JNO. T. TOLAND,
38, West Fourth street,
Cincinnati.

---

### Miscellaneous.

| | | |
|---|---|---|
| Steel Rings for Drills, each, | | 50 |
| Saliva Pumps, for removing the Saliva in filling the Lower Teeth, | | 1 00 |
| Bibulous Paper, for drying out cavities, per quire, | | 25 |
| Plug Plyers, for carrying the Gold to the cavity, | | 50 |
| Plain Socket, for Nerve Bitts, Excavators, etc., | | 50 to 1 00 |
| Bitts, for extracting Nerves, per dozen, | | 75 to 1 50 |
| Burrs, Drills and Excavators for Sockets, per doz., | | 75 to 1 50 |
| File Carrier, Ivory Handle, | | from 1 50 to 3 50 |
| do. Pearl do. | | 3 50 to 5 00 |
| Portable Head-rest, | | 6 00 |
| Pivot Wood, | per box, | 50 |
| Steel Shears, for cutting Gold Foil, | | 1 25 |
| Dentists' Account Books, Bronson & Bro. with index, | small, | 3 00 |
| do. do. do. | large, | 6 00 |
| do. do. other varieties, at various prices. | | |
| Floss Silk, per spool, | | 15 |
| Napkin Holder, | | 1 25 |
| Mouth Distender, for holding the mouth open, | | 50 |
| Cheek Holders, Pearl, | | 75 to 2 50 |
| Yellow Wax, | | 35 |
| White Wax, | | 75 |
| Hill's Stopping, | per oz., | 8 00 |
| Evans' Amalgam, | " | 2 00 |
| New do. composed of pure Tin and Silver, | " | 2 00 |
| do. do. *chemically* pure, | | 4 00 |
| Spittoon Bowls or Funnels, of Porcelain, | | 1 50 |
| do. do. Claret-colored Glass, large, | | 1 50 |
| Speltre, | per. ℔. | |
| Lead, | " | |
| Tin, in Bars, | " | |
| Sheet Lead for cutting Patterns, | " | 25 |
| Porcelain Slabs, 12 in. square, | | 1 50 |

| | | |
|---|---|---|
| Porcelain slabs, 16 in. square, | | $3 00 |
| Sponge Platina, | per dwt., | 63 |
| English Rose Red, best, | per oz., | 1 50 |
| Drs. Allen and Hunter's Silicious Compound for attaching Teeth to Plates, | per oz., | 50 |
| Gum Enamels, for same, | per oz., | 2 00 |
| Tooth Powder, | per lb., 1 00 to | 2 00 |

Tooth Brushes, from 50 to $4 00 per dozen.

| | | |
|---|---|---|
| Asbestos, | $ | 25 |
| Tannin, | per oz., | 50 |
| Morphia Sulphas, | per $\frac{1}{8}$ oz,, | 75 |

Dental Furnace, Nos. 1, 2 and 3, muffles and slides to suit.

Oxide Uranium.
" Titanium.
" Cobalt.
" Manganese.
" Gold.

| | | |
|---|---|---|
| Spar, prepared, | per lb., $ | 50 |
| Silex, " | " | 75 |
| Kaolin, " | " | 25 |
| Block Body, | " | 2 50 |
| Gum Enamel, for ditto, | per oz., | 2 00 |
| Plain " for point and base, | " | |

Plaster Paris, a superior article, price per quart 10c, per bushel $2 00, per bbl. $4 50.

White Sand, 5 cents per quart.

Moulding Sand, 5 cents per quart.

Crucibles, 10 cents per nest.

---

### Periodicals.

| | |
|---|---|
| Dental Register of the West, (Quarterly,) | $3 00 |
| Dental News Letter, | 1 50 |
| American Journal of Dental Science, | 5 00 |
| Dental Obturator, | 2 00 |

---

### Chevalier's Portable Lathe.

Price $15. Do. with extra heavy wheel, $16 to $18. Burs and Saws for Lathe, 50 cents each.

### Mouth Distender.

Above we give a cut showing a new instrument and its application. By its use a more thorough examination of the mouth may be made, than with any other appliance we have ever seen, and it may be used also with great advantage in filling the back teeth, by having the patient hold it in position. The material is Britannia metal. Price 50 cents.

---

### Hand Lathes.

Above are views of three hand Lathes, all accelerated motion. Prices $3, $3 and $4.

This small Lathe, occupying a space 6 inches by 4 inches, will be found very convenient and effective for those having to perform operations out of the office. Price $6 50.

### Mead's Lathe.

This machine is designed to be screwed to a bench or a table, and carries two corundum wheels at a time, or a wheel and brush if desired. A is a cast iron rest, moveable at the will of the operator; B is one of the corundum wheels, and C the other. D is the attachment for movement by the foot, and E the handle when hand power is employed. The balance-wheel is proportioned to the size and power of the apparatus. This is the best grinding machine with which we are acquainted, at the price, suitable for either a stationary or traveling dentist. Price $10.

### Locke's Flask.

No. 1.

No. 2.

No. 3.

No. 4.

This "casting apparatus" is composed of four parts which we shall designate by numbers thus: Moulding Flask No. 1; Follow Plate, No. 2; Die Flask, No. 3, and Counter Die Flask, No. 4.

MODE OF USING THEM.—After the impression is prepared for receiving the plaster, and any excess of the outer rim removed, it is filled with plaster, when No. 2 is adjusted, with the convex side down, upon it, and a little more plaster filled in the concavity of No. 2, to fasten it to the plaster cast beneath. Care should be taken to adjust No. 2 so that the plaster cast will be in the center of it when removed from the impression.

After removing, there will be found on the face of No. 2, a plaster cast only of the palatial and alveolar ridge; and, consequently, but little to trim, varnish and mould.

After trimming and varnishing, place No. 2 upside down on a board, and No. 1 also upside down over it, and the face of the cast will be right for packing the sand. Fill up No. 1, and pack well; then turn upside down, after tapping it lightly on the side, and you will usually find the cast attached to No. 2 where you placed it on the board. If not, remove in your usual way. Then place No. 3, with large end down, over the impression in the sand, when it will perfectly fit into the depression made by the flange on the edge of No. 2, as well as to the opening in No. 1.

The first of the metal is poured into the little cup in No. I, behind No. 3, for the purpose of filling up the bottom without displacing the sand—balance poured into the top of No. 3. Price, $1 50 each—set of four pieces.

After cooling and cutting off the little protuberance with a chisel, on the back of the die, and preparing the face of it in the usual way, place the larger end of No. 4 over it, and proceed to fill up the counter die.

### Hawes' Moulding Flask.

Fig. 1.

Fig. 2.

Fig. 3.

Fig. 1 represents the lower section of the Flask, slightly opened, to show the joints. Fig. 2 is the upper section. When ready for use, the lower section is closed and confined by a pin and the plaster model placed in it, as represented in Fig. 3. Price, $2.

---

### Luther's Moulding Flask.

A neat, convenient and useful apparatus, by which much time and labor is saved and difficulty avoided, requiring but little sand or plaster. Price, $1.

---

### Works on Dentistry.

| | |
|---|---|
| Harris' Principle and Practice of Dental Surgery, 6th Edition, | $4 00 |
| Harris' Dictionary of Dental Surgery, | 4 50 |
| A treatise on the Disease and Surgical Operations of the Mouth, by M. Jourdain, | 2 25 |
| A Practical Treatise on Dental Medicine, by Thos. E. Bond, M. D., 2d Edition, | 2 50 |
| Treatise on the Diseases of the Teeth, by Robt. Arthur, D. D. S., | 50 |
| Ether and Chloroform, their use in Surgery, Dentistry, &c., by J. F. B. Flagg, M. D., | 75 |
| Piggott's Dental Chemistry and Metallurgy, | 3 00 |

| | |
|---|---|
| Fox & Harris, on the Human Teeth, . . . . | 4 00 |
| Tomes' Dental Physiology and Surgery, . . . . | 3 50 |
| Handy's Text and Book of Anatomy, . . . . | 3 00 |
| Wilson's Anatomy, . . . . . . . . . | 3 00 |
| Carpenter's Physiology, . . . . . . | 4 25 |
| Williams' Principles of Pathology, . . . . . | 2 25 |

I am also prepared to supply any other books, Dental or Medical, at the publisher's prices.

---

### Mallett's Adjustable Punch Forceps.

This improvement consists in making an Adjustable Punch, so that they shall punch the holes in the plate to exactly correspond with the pins in the teeth. It includes the combination of two punches, one immovable and the other movable in the slot, (*h*,) with a spring, (*g*,) and the two cavities, one (*i*,) in the plug or immovable Punch, and the other, (*h*,) movable with a movable Punch; the latter to be set by the inserting of the wires of the teeth into the cavities, in order to make the distances between the holes in the plate correspond with the distances of the pins in the teeth, as described.

To use this Punch, we place one of the pins of the teeth into the hole, (*i*,) and force out the movable Punch far enough to allow the other pin in the tooth to pass into the slot, (*h*,) which will adjust the two punches to exactly the distance of the pins, so that when the plate is punched the holes in the plate will receive the pins with perfect accuracy in every case. Price, $5.

### Franklin's Improved Under-Impression Cup, for Plaster Impressions.

UPPER SIDE.

UNDER SIDE.

DIRECTIONS: Fill the under part of the cup with plaster, mixed to a consistency, so as to keep its position in the cup, and the upper part half full. Place the cup in the mouth as expeditiously as possible, and gently press it down as far as desired. The cup should then be firmly held in its position, the operator pressing with the ends of the fingers upon the plaster in the upper part of the cup, forcing it down through the space, which will insure a most perfect impression. After the plaster is sufficiently set, (*which requires from four to eight minutes,*) the cup may be gently removed from the mouth, the impression oiled and filled in the usual manner. After the filling has become sufficiently hard, the plaster in the upper part of the cup should be cut away, down to the space or bottom of the upper part of the cup, and all the surplus plaster that adheres to the outside, should be cut away, when the cup may be easily removed from the mass. Then warm the plaster to about blood heat, when the impression may be broken away from the model.

The advantage of this cup over all others in use—*independent of its superior shape and adaptation*—is in having a surplus of plaster to be acted upon after the cup is placed in the mouth and brought to its proper position, preventing the occurrence of any blanks or other imperfections in the impression. Price, $1.

---

### Caution.

Efforts having been made to palm off as "Sherwood's patterns" Forceps made by inferior workmen, I would state, for the benefit of the profession, that all Forceps made by Mr. Sherwood are *stamped* on the joint H. R. Sherwood on one side, and on the other side Jno. T. Toland.

### Dental Furnaces.

On this page is a drawing of a furnace designed, and manufacture for baking blocks and Continuous Gum work. The two upper muffles are for heating and annealing the work, the blaze and heat from the fire passing between and around them. They are made in a substantial manner, two inches thick, well hooped, and tapering or larger below, to admit of the coal settling around the lower or baking muffle.

The two-story furnace, with three muffles, doors, stoppers and muffle-rest, $20; the one-story furnace, with muffle, doors, stoppers, and muffle-rest, $14. Boxing and packing extra.

---

### A Little Plain Talk!

Having been informed that, as a last effort to MONOPOLIZE the Western Dental Trade, and to drive me out of the competition, TEETH and other Goods are sent to *prominent* points in the country on *commission* to Dentists, to be sold or used, and accounted for at stated periods for the amount sold or consumed, I wish to make a few remarks concerning this mode of doing business.

Why is this system adopted *now*, after ten years' persistent refusal of scores of applicants, on the ground that the profits would not pay the interest on the stock? Would it be done *now*, if there was no competition? How long would it continue, if the competition was forced (for want of support) to discontinue?

Being unprofitable, it will be continued only so long as to *effect* the object. Should it fail to accomplish the object desired, it will cease because of its being unprofitable. Of necessity, it must, therefore, be temporary! Is it desirable to the Dentist to enjoy a *temporary* advantage at the *sacrifice* of a permanent one? Is it reasonable to suppose that an *agent* can afford to do what the manufacturers cannot?

Being a *beginner*, with *small capital*, under heavy expenses, and a rapidly increasing trade, to properly and *promptly* accommodate which, it is necessary for me to keep a large stock on hand, my means will not justify me in dividing my stock, to *benefit* a FEW, to the *injury* of the MANY. I prefer, therefore, to keep all my stock at home, that the *whole profession* may be *equally* and *well accommodated.*

To those who will *purchase* in large quantities, I will sell at *prices* sufficiently *low* to more than counterbalance the advantages of an agency.

---

### Local Anasthesia.

This is a neat and handsome instrument, invented and patented by Dr. I. B. Branch, of Galena, Ill. Although it is patented, the terms are liberal, and the price as low as any that have been in the market, not patented. Price of instrument and office right, $10.

Having almost daily inquiries relative to this invention, I publish the following, being a circular letter issued by Dr. Branch, to which are attached nine or ten *certificates* of respectable Dentists, Physicians, etc.

Dear Sir:—I send you this to answer the inquiries which correspondents are constantly making, concerning my Instruments and Agent for the production of Local Insensibility to Pain during Dental and Surgical Operations. These questions are,

1st.—Are they efficient? To this I answer that, in extracting Teeth, they do produce the effect so perfectly that, in a *large majority* of cases, the operation is entirely painless, and, in all cases, it so mitigates the suffering as to render it a comparatively trifling matter.

The second query is—Are they harmless? I answer, in proper hands with proper instructions, they are absolutely so. I have used them, in hundreds of instances, and other Dentists, some of whose names are attached to this, have done the same, with no ill consequences. No pain follows the operation in cases of acute periostitis, such as is common when the operation is performed without its use, and the gums appear to heal with less inflammation and swelling than in ordinary cases without it.

3d query.—Are they portable? Yes; they may be carried in the pocket, or sent by mail or express.

4th.—How much time is consumed in producing the effect? I answer, $1\frac{1}{2}$ to 5 minutes after the application is made. The effect is confined to the immediate vicinity of the operation, producing no mental or constitutional results.

I. B. BRANCH, Galena, Ill.

Agent for Cincinnati: JNO. T. TOLAND,
38 *West Fourth st.*

---

### Crystaline Gold.

The enquiries relative to the merits of this article are so numerous, that we are constrained to make a few remarks on the subject.

That good fillings can be made of Crystal Gold is no longer a subject of doubt, we have the evidence of some of the best Dentists in the country in its favor. Some of the finest plugs we have ever seen were made of this preparation, but whilst a few have been eminently successful, the majority have utterly

*failed!* This has not been owing to want of skill on the part of the operator, but partly for the want of proper instruments and partly dependent on the *Gold itself.*

About two years ago a quantity of Crystal Gold was put in market, which done more injury to those who used it than they will be able to *retrieve* in years.

We have seen this illustrated in the mouths of three different patients—our intimate friends. The operations were performed by one of the best Dentists in this city, the fillings were beautiful, full, solid and smooth as a piece of burnished plate, commanding the admiration of all who saw them.

But unfortunately, in a short time a dark ring appeared around the margin of the fillings, and finally the plugs dropped out entire, leaving the teeth in much worse condition than before the operation. Several of the teeth were frail before—the decomposition of bone around the filling so weakened them, that they crumbled away and were entirely lost, when they could have been saved with foil. The difficulty seems to be in not having the Gold entirely *freed* from the CHEMICAL agents used in its manufacture, the tooth bone having a strong affinity for acids, the slightest trace would be sufficient to *destroy* the *tooth.*

The manufacturers have promised that the like should not occur *again;* but human nature is not perfect, the same accident which occurred once may occur again. No Dentist can take the time and trouble to test each box of Gold; in fact, many have not the convenience for so doing. Besides, where is the use, when precisely the same kind of work can be done with good Foil *annealed* and worked with proper instruments.

In fact, the great secret of working the Crystal Gold is in the INSTRUMENTS, these are just as *valuable* for working *Foil* as Crystal.

## Figures and Tables

**Figure f1:**
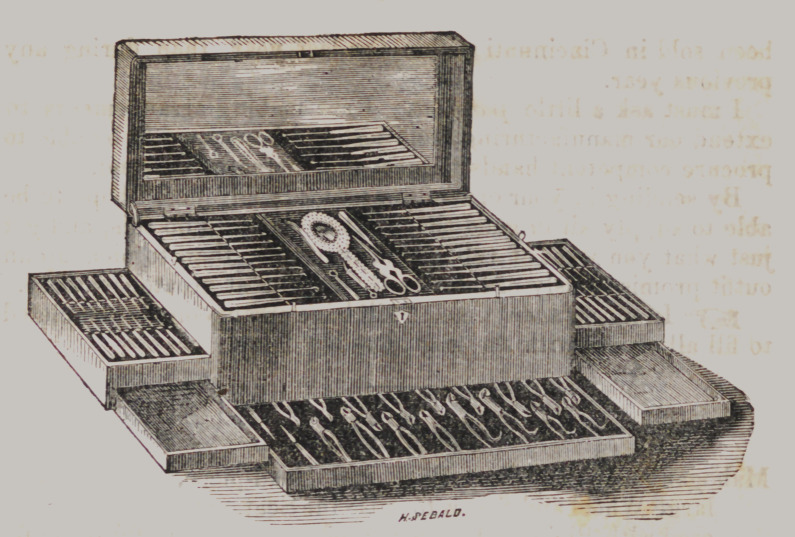


**Figure f2:**
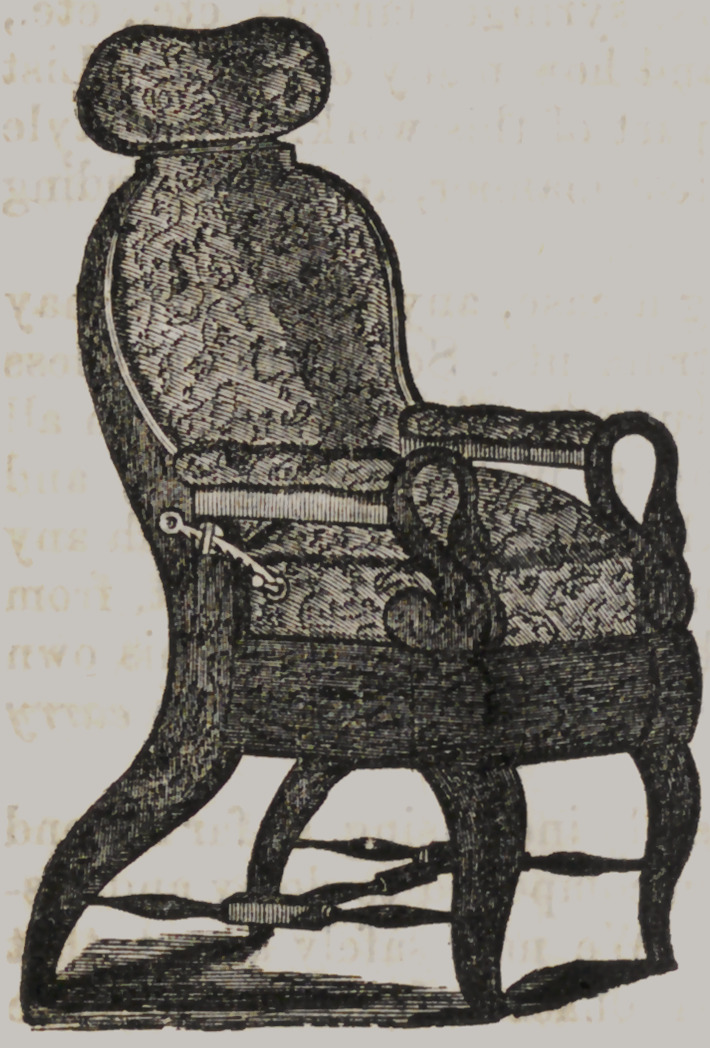


**Figure f3:**
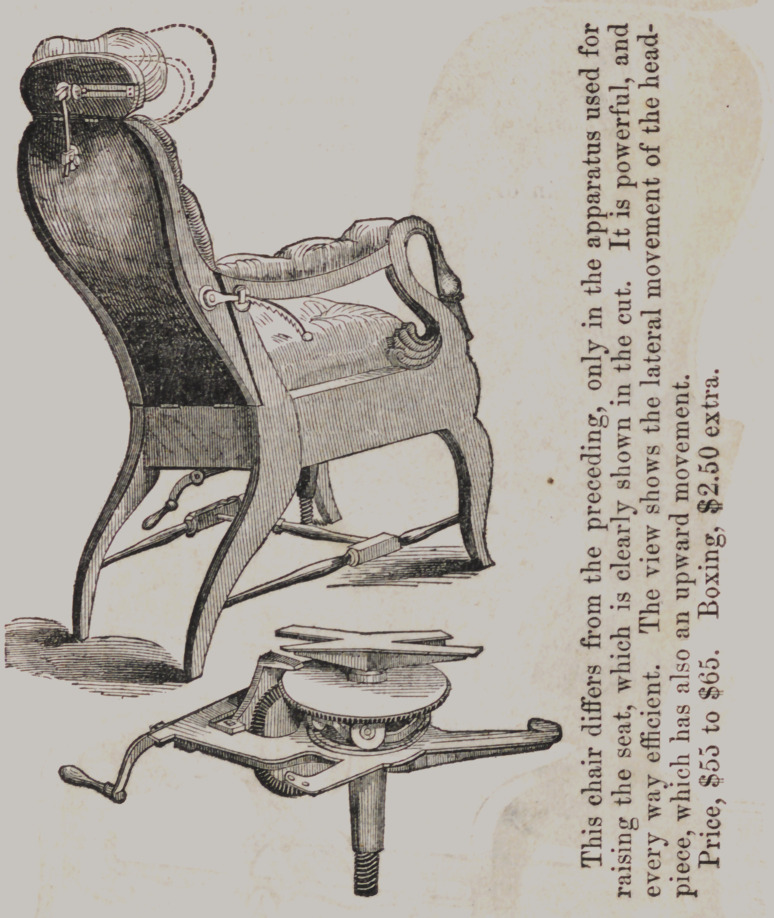


**Figure f4:**
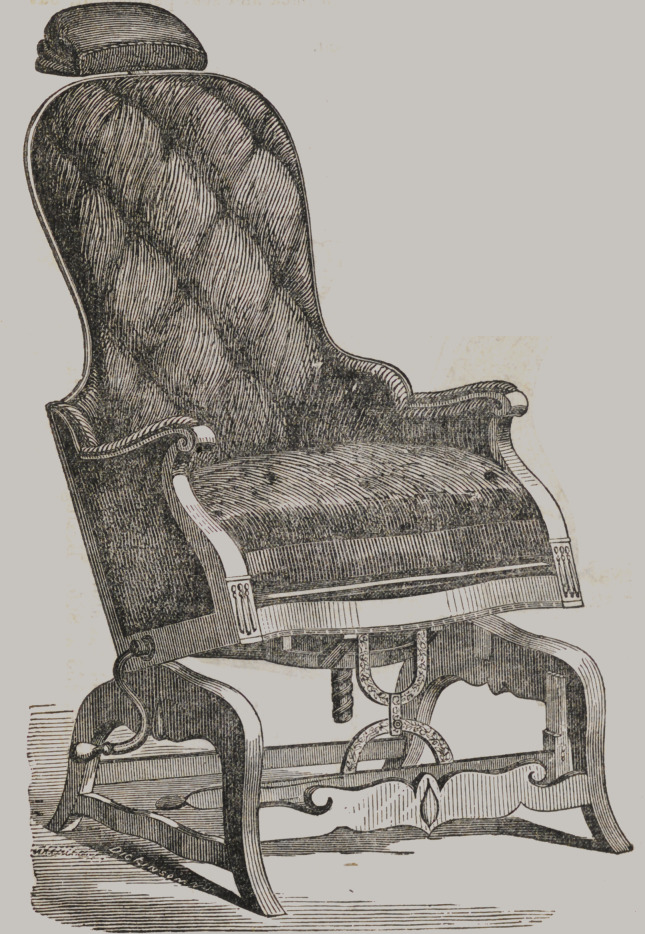


**Figure f5:**
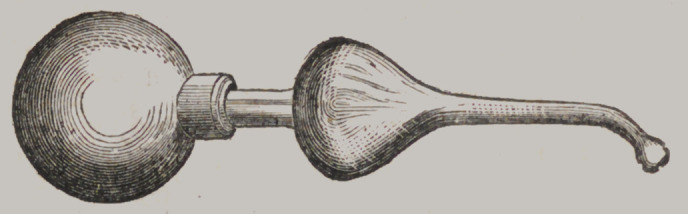


**Figure f6:**
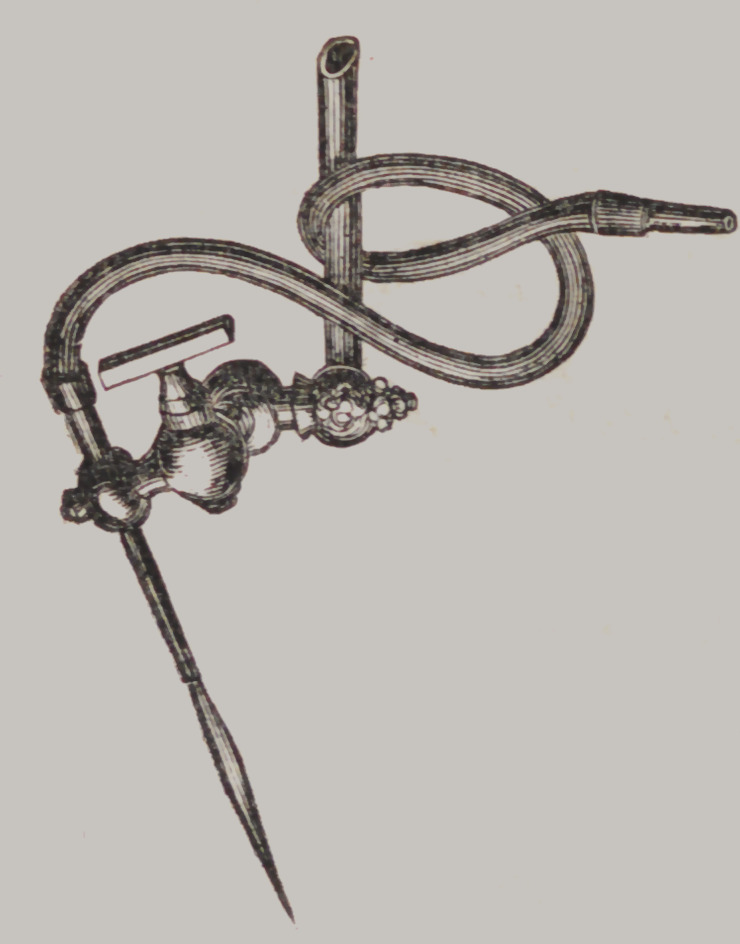


**Figure f7:**
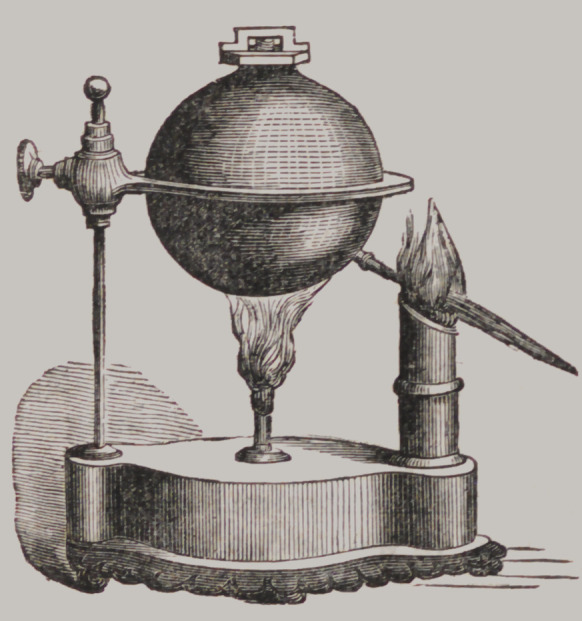


**Figure f8:**
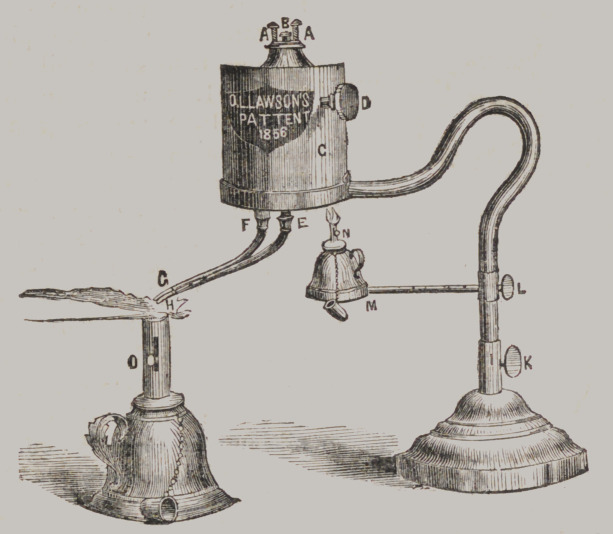


**Figure f9:**
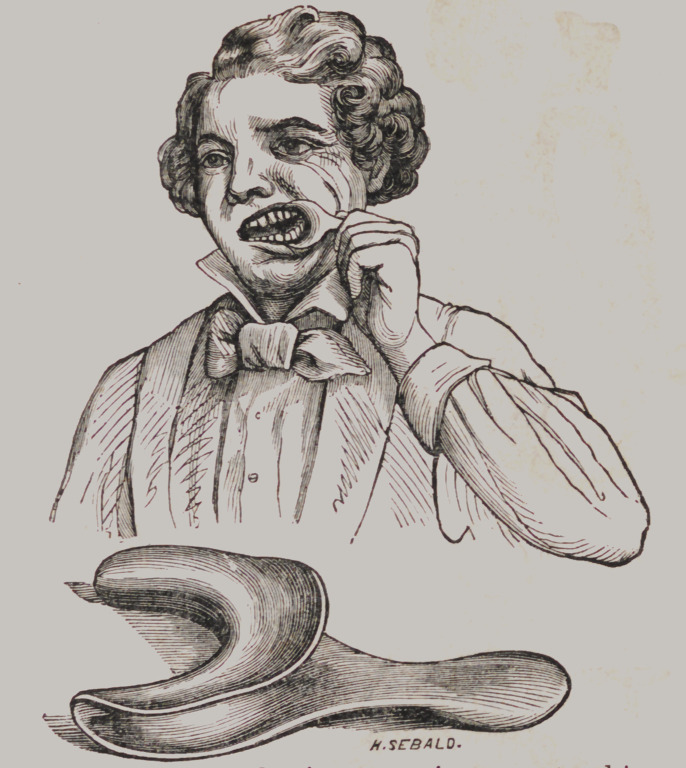


**Figure f10:**
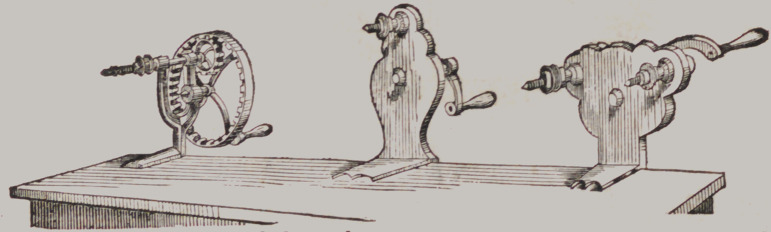


**Figure f11:**
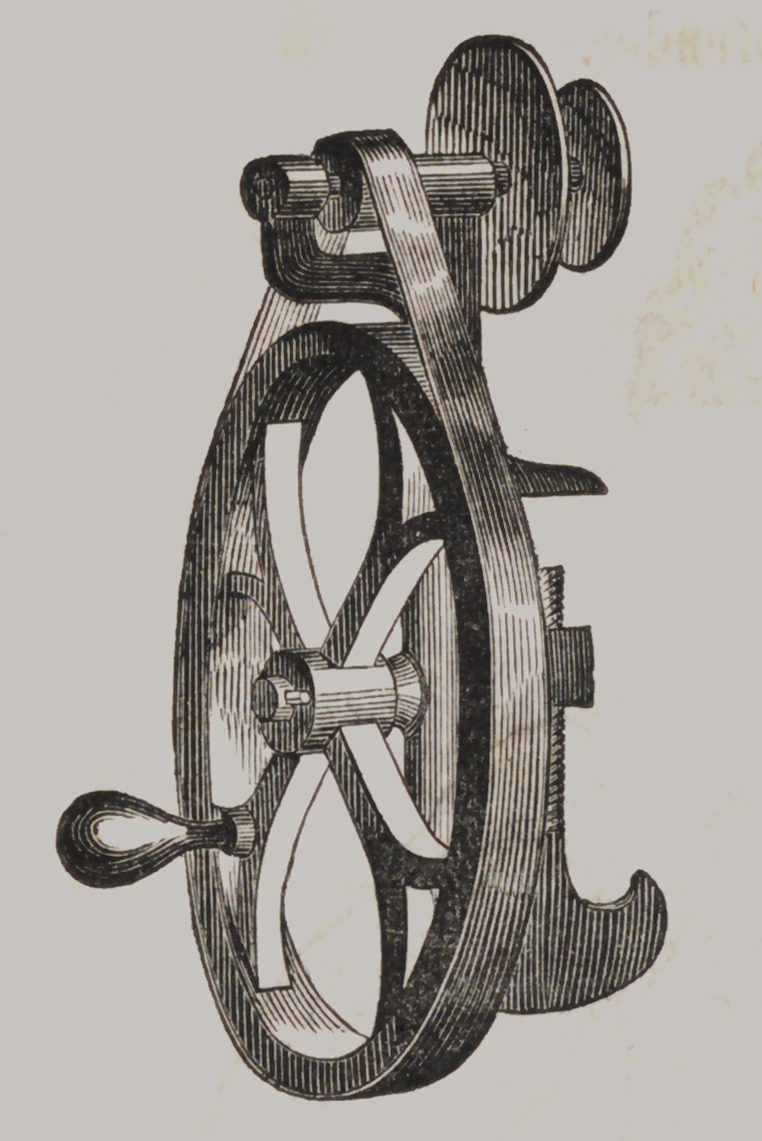


**Figure f12:**
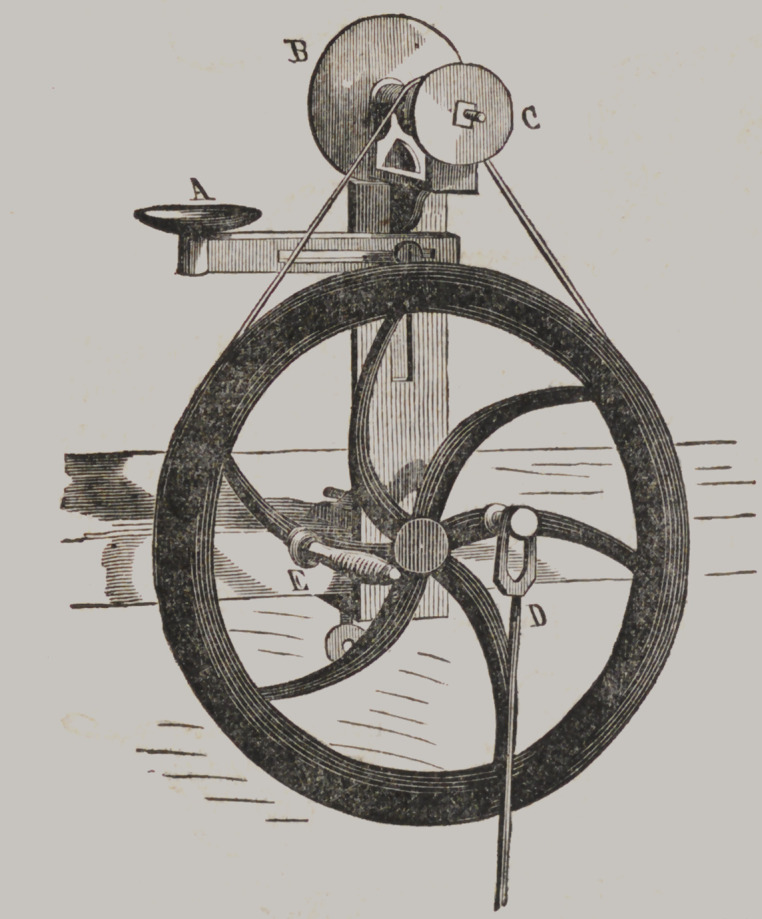


**No. 1. f13:**
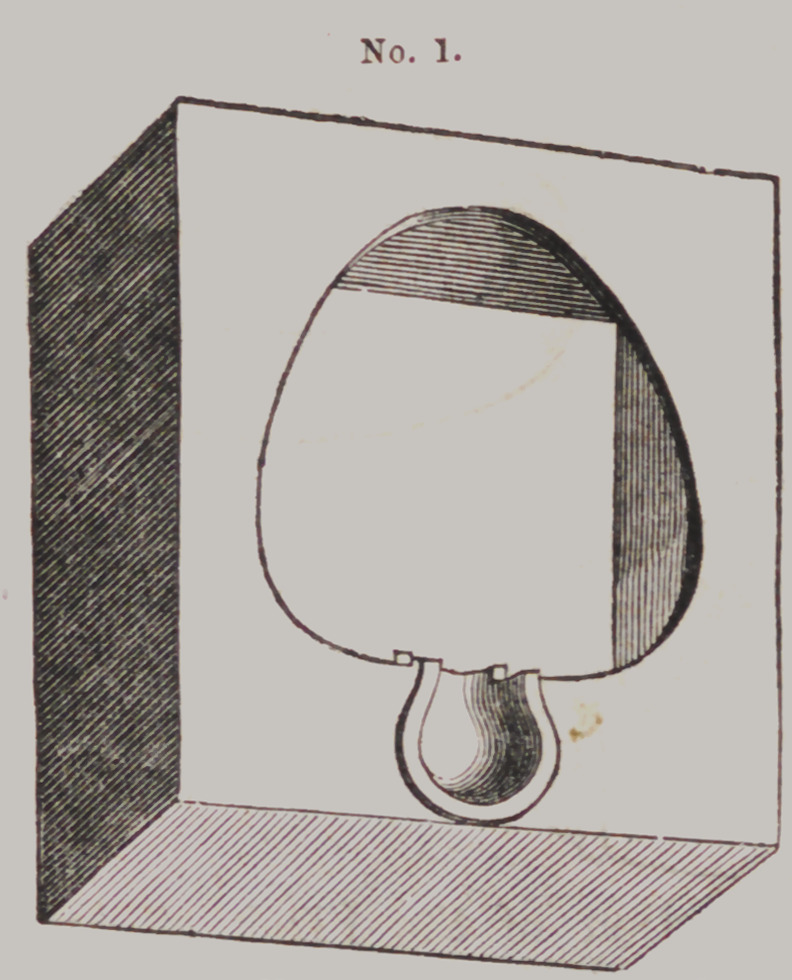


**No. 2. f14:**
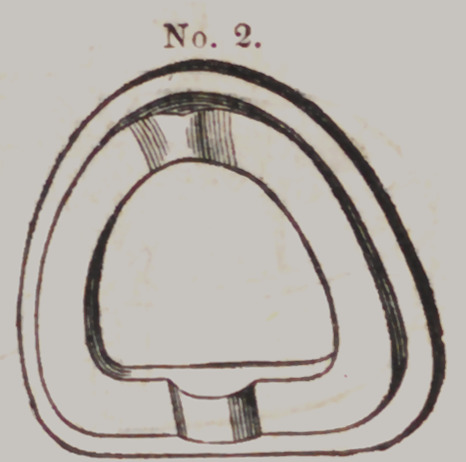


**No. 3. f15:**
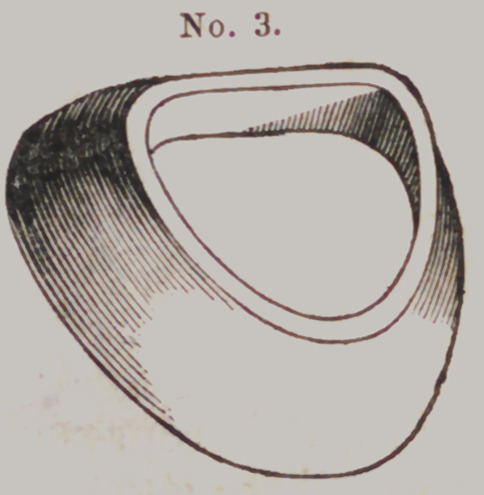


**No. 4. f16:**
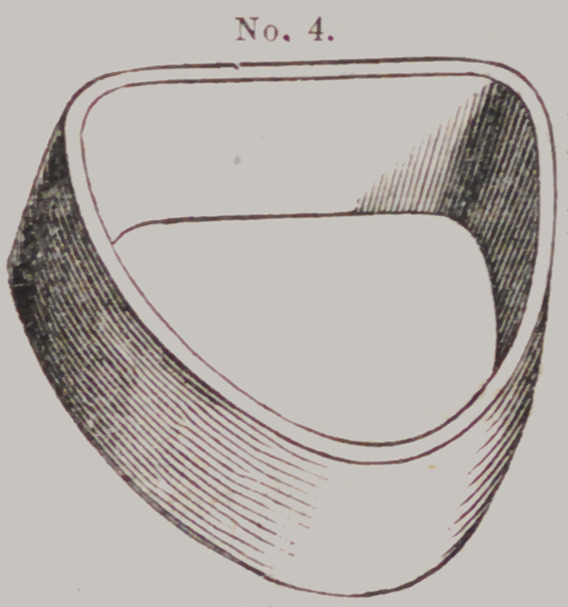


**Fig. 1. f17:**
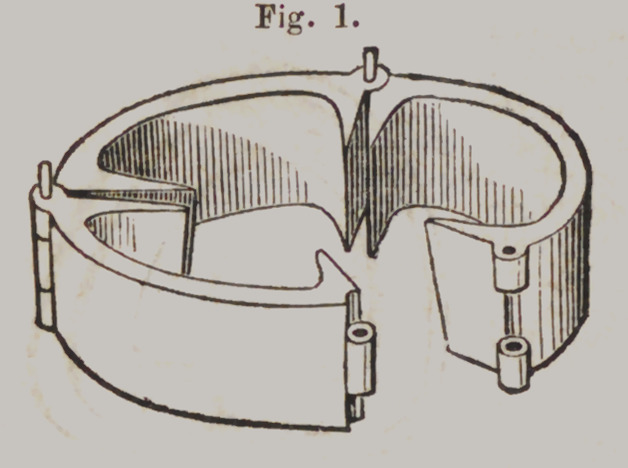


**Fig. 2. f18:**
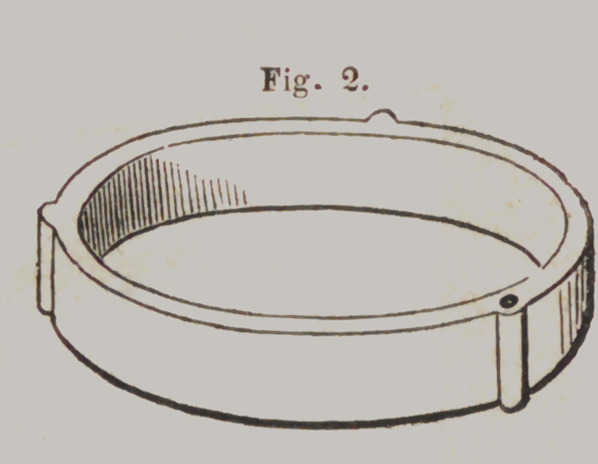


**Fig. 3. f19:**
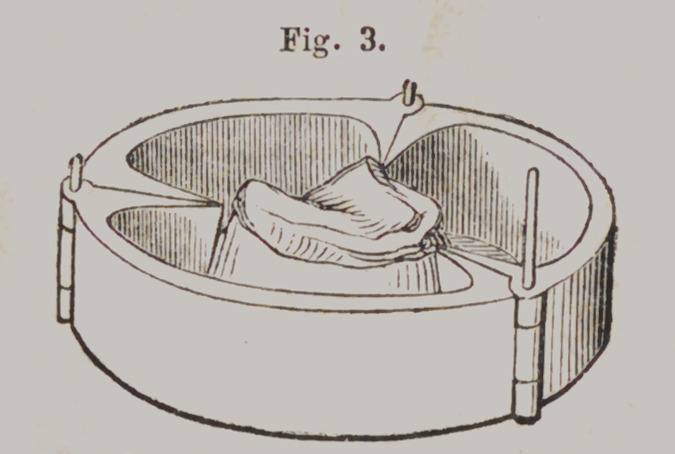


**Figure f20:**
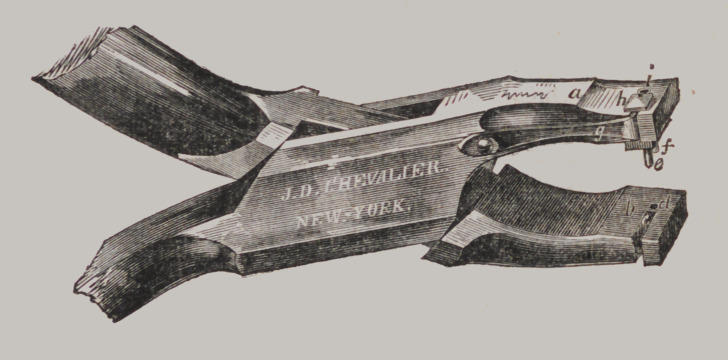


**Figure f21:**
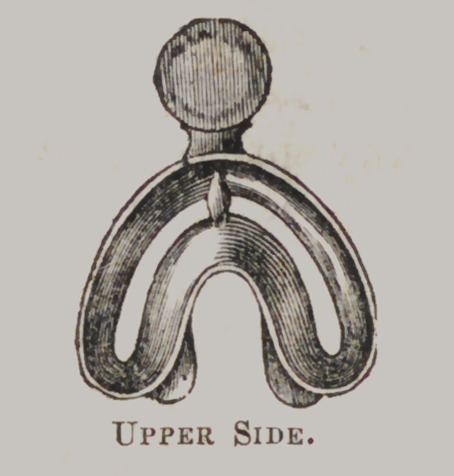


**Figure f22:**
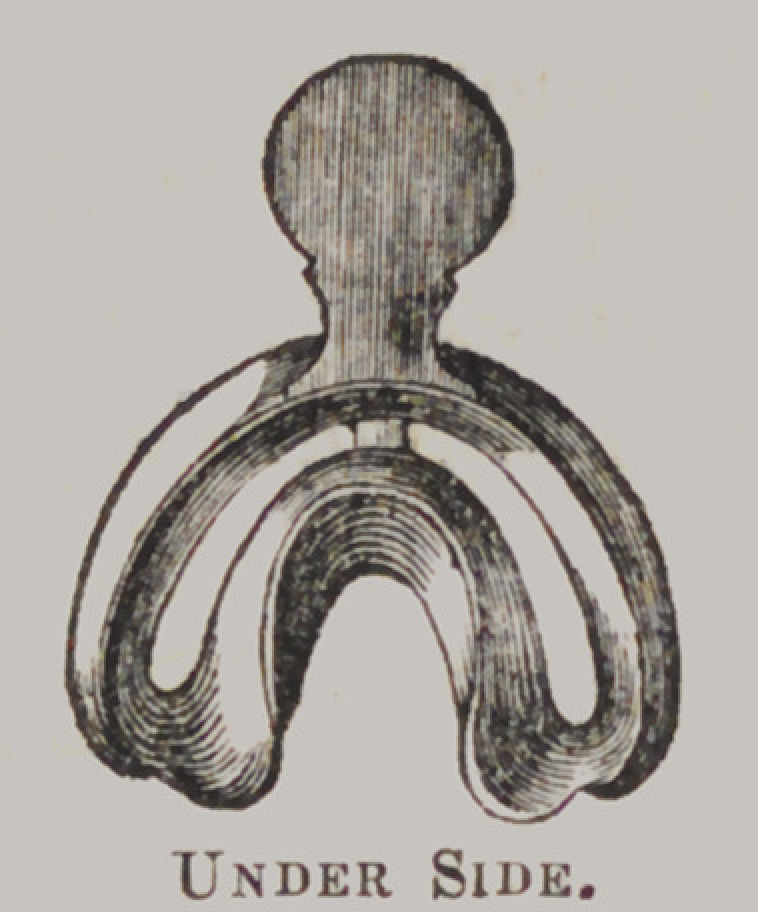


**Figure f23:**